# High Spatiotemporal Near‐Infrared II Fluorescence Lifetime Imaging for Quantitative Detection of Clinical Tumor Margins

**DOI:** 10.1002/advs.202411272

**Published:** 2024-12-09

**Authors:** Zhen Chen, Linjian Huang, Duyang Gao, Zhouzhou Bao, Dehong Hu, Wei Zheng, Jing Chen, Jiuling Liao, Hairong Zheng, Zonghai Sheng

**Affiliations:** ^1^ State Key Laboratory of Radio Frequency Heterogeneous Integration College of Electronics and Information Engineering Shenzhen University Shenzhen 518060 P. R. China; ^2^ Research Center for Advanced Detection Materials and Medical Imaging Devices Paul C. Lauterbur Research Center for Biomedical Imaging Institute of Biomedical and Health Engineering Shenzhen Institute of Advanced Technology Chinese Academy of Sciences Shenzhen 518055 P. R. China; ^3^ Institute of Microscale Optoelectronics Shenzhen University Shenzhen 518060 P. R. China; ^4^ Research Center for Biomedical Optics and Molecular Imaging Shenzhen Key Laboratory for Molecular Imaging Guangdong Provincial Key Laboratory of Biomedical Optical Imaging Technology Key Laboratory of Biomedical Imaging Science and System Shenzhen Institute of Advanced Technology Chinese Academy of Sciences Shenzhen 518055 P. R. China; ^5^ Department of Obstetrics and Gynecology Shanghai Key Laboratory of Gynecologic Oncology Ren Ji Hospital School of Medicine Shanghai Jiao Tong University Shanghai 200127 P. R. China

**Keywords:** indocyanine green, molecular target, near‐infrared‐II fluorescence lifetime, tumor margin

## Abstract

Accurate detection of tumor margins is essential for successful cancer surgery. While indocyanine green (ICG)‐based near‐infrared (NIR) fluorescence (FL) surgical navigation enhances the visual identification of tumor margins, its accuracy remains subjective, underscoring the need for quantitative approaches. In this study, a high spatiotemporal fluorescence lifetime (FLT) imaging technology is developed in the second near‐infrared window (NIR‐II, 1000–1700 nm) for quantitative tumor margin detection, utilizing folate receptor‐targeted ICG nanoprobes (FPH‐ICG). FPH‐ICG exhibits a significantly prolonged NIR‐II FLT (750 ± 7 ps vs 260 ± 3 ps) and increased NIR‐II FL brightness (FPH‐ICG/ICG = 3.8). In vitro and in vivo studies confirm that FPH‐ICG specifically targets folate receptor‐α (FRα) on SK‐OV‐3 ovarian cancer cells, achieving high‐contrast NIR‐II FL imaging with a signal‐to‐background ratio of 10.8. Notably, NIR‐II FLT imaging demonstrates superior accuracy (90%) and consistency in defining tumor margins compared to NIR‐II FL imaging (58%) in both SK‐OV‐3 tumor‐bearing mice and clinical tumor samples. These findings underscore the potential of NIR‐II FLT imaging as a quantitative tool for guiding surgical tumor margin detection.

## Graphical abstract

Folate receptor‐targeted indocyanine green (ICG) nanoprobes are designed for quantitative tumor margin detection utilizing fluorescence lifetime (FLT) imaging technology in the second near‐infrared window (NIR‐II). The NIR‐II FLT imaging exhibits enhanced accuracy (90%) and consistency in delineating tumor margins compared to NIR‐II fluorescence imaging (58%) in both mice model and clinical tumor specimens.

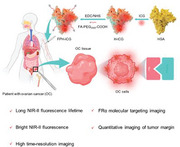

## 1 Introduction

Surgery remains the primary treatment modality for most solid tumors.^[^ ^1^ ^]^ The success of surgical interventions is highly dependent on the accurate delineation of tumor margins.^[^ ^2^ ^]^ Intraoperative near‐infrared (NIR) fluorescence (FL) imaging in combination with various dyes^[^ ^3^ ^]^ such as indocyanine green (ICG) is increasingly being used to illuminate cancer cells and improve the visibility of tumor margins.^[^ ^4^ ^]^ Recent studies have shown that ICG emits tail FL in the second near‐infrared window (NIR‐II, 1000–1700 nm),^[^ ^5^ ^]^ a transparent region characterized by lower tissue absorption,^[^ ^6^ ^]^ scatter,^[^ ^7^ ^]^ and background FL compared to the first near‐infrared window (NIR‐I, 650–950 nm).^[^ ^8^ ^]^ These unique advantages have enabled significant advances in the precise delineation of tumor margins during surgery.^[^ ^9^ ^]^ However, the inherently low NIR‐II FL ICG brightness,^[^ ^10^ ^]^ the lack of specific molecular targeting for cancer cells,^[^ ^11^ ^]^ and the dependence on various imaging parameters still hinder the accurate quantification of tumor margins.^[^ ^12^ ^]^

In contrast to FL imaging, fluorescence lifetime (FLT) ICG imaging can better reflect its intrinsic molecular properties and the characteristics of the surrounding microenvironment,^[^ ^13^ ^]^ offering advantages that are independent of imaging parameters and environmental variations.^[^ ^12^ ^]^ Consequently, ICG FLT can detect microenvironmental differences between cancer cells and normal cells, such as variations in cellular viscosity, thus facilitating the quantification of the surgical tumor margin.^[^ ^14^ ^]^ Recent findings by Pal et al. indicate that ICG has a longer FLT in tumor tissues compared to normal tissues in several cancer types, suggesting its potential as a universal marker for tumor margin identification, thereby improving surgical precision.^[^ ^14^ ^]^ Despite these advances, the application of ICG FLT for molecular margin visualization is limited by the lack of tumor‐specific targeting of free ICG, which cannot identify receptors overexpressed on cancer cell surfaces. In addition, existing ICG FLT techniques typically operate within the NIR‐I window, limiting the quality of deep tissue imaging.^[^ ^15^ ^]^

**Scheme 1:**
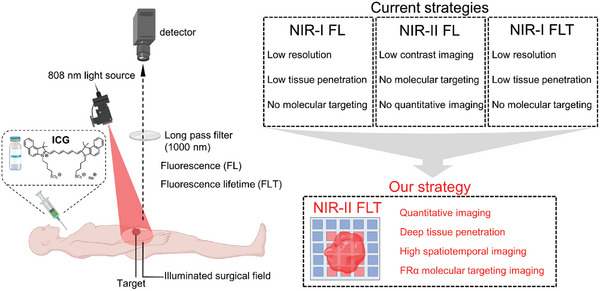
Scheme of near‐infrared‐II (NIR‐II) fluorescence lifetime (FLT) imaging of clinical tumor margins.

In this study, we report for the first time on the use of engineered folate receptor‐α‐targeted ICG nanoprobes (FPH‐ICG) for NIR‐II FLT imaging of clinical tumor molecular margins (**Scheme** **1**). Molecular docking combined with polyethylene glycol (PEG) engineering was employed in the design and preparation of FPH‐ICG to ensure uniform size, high NIR‐II FL brightness, stable NR‐II FLT, and effective targeting of folate receptor‐α (FRα). We have systematically evaluated the FRα targeting ability of FPH‐ICG in both preclinical and clinical models and demonstrated its superiority in quantitatively determining the tumor margin compared to NIR‐II FL imaging. We also conducted studies in ovarian tumor‐bearing mouse models using FPH‐ICG NIR‐II FLT‐guided precision surgery, demonstrating the potential of the technique to improve surgical outcomes. These results demonstrate that NIR‐II FLT can improve the delineation of tumor margins in vivo and in vitro. This technique could potentially reduce local recurrence following surgery.

## 2 Results and Discussion

### 2.1 Preparation and Characterization of FPH‐ICG

**Figure 1:**
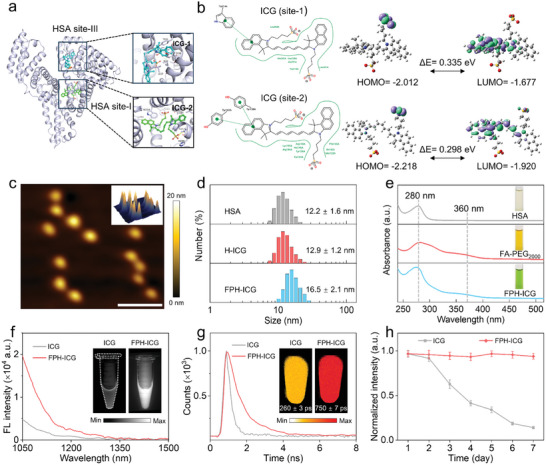
Characterization of FPH‐ICG. a) Molecular docking model showing two binding sites of ICG within HSA. The enlarged view within the dashed box highlights the specific binding details of ICG within the HSA hydrophobic pocket. b) Illustration of the frontier molecular orbitals (LUMOs and HOMOs) determined at the B3LYP/6‐31G(d) theory level. c) AFM image of the FPH‐ICG with a 3D height image in the inset. Scale bar = 0.3 µm. d) Hydrodynamic size distribution of HSA, H‐ICG, and FPH‐ICG. e) Absorption spectra of HSA, FA‐PEG_2000_, and FPH‐ICG from 240 to 515 nm. f) FL spectra of ICG and FPH‐ICG in the NIR‐II region. Inset: FL images of ICG and FPH‐ICG solutions. ICG concentration: 5 µg mL⁻¹, excitation wavelength: 808 nm, 1000 nm long‐pass filter, exposure time: 10 ms. g) FL decay profiles of ICG and FPH‐ICG in the NIR‐II region. Inset: FLT images of ICG and FPH‐ICG solutions. ICG concentration: 25 µg mL⁻¹, excitation wavelength: 808 nm, 1000 m long‐pass filter. h) FL stability of ICG and FPH‐ICG solutions over a 7‐day storage period.

The design and preparations of FPH‐ICG involved molecular docking analysis to assess the interaction between albumin and ICG, followed by rational modification with PEG_2000_‐folate (PEG_2000_‐FA) (Figure S1, Supporting Information). The molecular docking results showed that albumin can form stable protein‐dye complexes (H‐ICG) with two ICG molecules (**Figure** **1**a).^[^ ^5^ ^]^ The quantitative calculation indicated that the LUMO and HOMO Egaps of ICG bound to site‐I (0.335 eV) and site‐II (0.298 eV) were lower than that of the free ICG (2.230 eV), suggesting that ICG embedding in albumin promotes molecular excitation (Figure 1b; Figure S2, Supporting Information).^[^ ^16^ ^]^ Further modification with PEG_2000_‐FA increased the stability of the H‐ICG and conferred its ability to target the folate receptor.^[^ ^17^ ^]^ Atomic Force Microscopy (AFM) images revealed a uniform spherical morphology with a mean diameter of 13.9 ± 0.5 nm (Figure 1c; Figure S3, Supporting Information). Dynamic Light Scattering (DLS) measurements indicated a larger hydrodynamic (16.5 ± 2.1 nm) for FPH‐ICG than that observed by AFM (13.9 ± 0.5 nm) due to the PEG modification on the H‐ICG surface. The hydrodynamic size (16.5 ± 2.1 nm) and zeta potential (−3.32 mV) of FPH‐ICG were higher than those of the H‐ICG nanoparticles (NPs) (12.9 ± 1.2 nm, −14.57 mV) (Figure 1d; Figure S4, Supporting Information), confirming the successful conjugation of PEG_2000_‐FA to the H‐ICG surface. Optical analysis revealed the characteristic absorption peaks of FPH‐ICG at 280 nm (albumin) and 360 nm (PEG_2000_‐FA) (Figure 1e), confirming the presence of PEG_2000_‐FA on the H‐ICG surface.^[^ ^18^ ^]^ FPH‐ICG NPs exhibited a red‐shifted NIR‐I FL emission peak at 813 nm compared to free ICG (800 nm), with enhanced emission in the NIR‐II window (Figure 1f; Figure S5, Supporting Information). In addition, the absorption peak of FPH‐ICG was shifted from 780 nm (free ICG) to 803 nm (Figure S6, Supporting Information), indicating an increased extinction coefficient. Notably, the NIR‐II FLT of FPH‐ICG was 750 ± 7 ps, nearly three times longer than that of free ICG (260 ± 3 ps) (Figure 1g),^[^ ^19^ ^]^ suggesting improved photophysical properties due to ICG encapsulation within the albumin hydrophobic domains.^[^ ^5^ ^]^ Furthermore, compared to NIR‐II FL properties, NIR‐II FLT of FPH‐ICG eliminated the influence of sample concentration and imaging physical parameters (exposure time and excitation power), thus resulting in more consistent imaging (Figure S7, Supporting Information). In addition, the optical stability assessments showed that FPH‐ICG maintained nearly unchanged NIR‐II FL intensity after 7 days of light‐protected storage at room temperature, whereas free ICG exhibited an 85% decrease in NIR‐II FL intensity under the same conditions (Figure 1h; Figure S8, Supporting Information). Taken together, these results demonstrate that FPH‐ICG has a long NIR‐II FLT and excellent optical stability, highlighting its potential for precise molecular imaging applications.

### 2.2 Active‐Targeting NIR‐II FLT Imaging In Vitro

**Figure 2:**
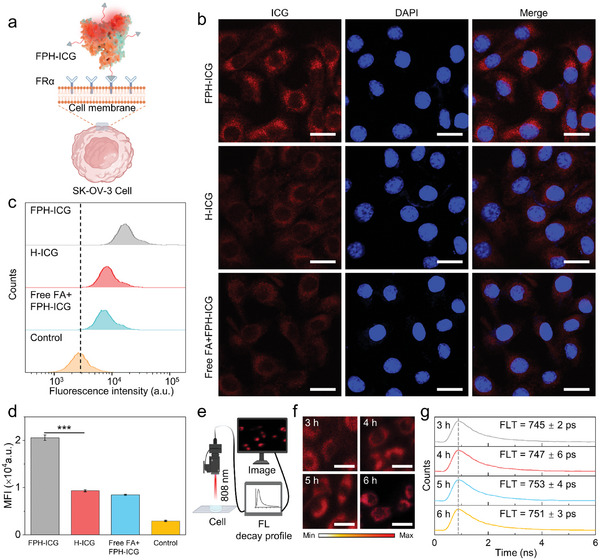
Cellular uptake of FPH‐ICG. a) Schematic representation of the phagocytosis process of FPH‐ICG. b) Confocal FL images of SK‐OV‐3 cells after being incubated for 3 h with either FPH‐ICG, H‐ICG, or free FA + FPH‐ICG. Red: ICG, blue: DAPI; scale bar = 30 µm. c) Flow cytometry analysis of cellular uptake of FPH‐ICG, H‐ICG, and free FA + FPH‐ICG. d) Quantitative analysis of the cellular uptake flow cytometry data. e) NIR‐II FLT imaging process of FPH‐ICG within cells. f) NIR‐II FLT images of FPH‐ICG using an excitation wavelength of 808 nm, long‐pass filter of 1000 nm, and an exposure time of 15 ms. The cells were cultured for 3 to 6 h. g) FL decay profiles of FPH‐ICG incubated with SK‐OV‐3 cells for 3, 4, 5, and 6 h. Data are presented as mean ± SD (*n* = 3). ***p < 0.001.

SK‐OV‐3 ovarian cancer cells overexpress the FRα receptor on their surface. This receptor is generally used as a molecular target to diagnose ovarian cancer (**Figure** **2**a).^[^ ^20^ ^]^ Therefore, we further optimized the number of folate modifications on the surface of FPH‐ICG to increase the efficiency of FPH‐ICG for active targeting of SK‐OV‐3 ovarian cancer cells. The flow analysis showed that the modified FPH‐ICG surface with four folate molecules resulted in the best targeting efficiency (Figure S9, Supporting Information). FL confocal microscopy imaging showed that significant FL of FPH‐ICG was observed in the cytoplasm of SK‐OV‐3 cells after 3 h of incubation. In contrast, both the H‐ICG‐treated group and the folate‐blocked group showed significantly lower FL intensity in SK‐OV‐3 cells compared to the FPH‐ICG‐treated group (Figure 2b). Quantitative flow cytometric analysis showed that FL signals in the FPH‐ICG‐treated group were 2.4‐fold and 2.2‐fold higher than those in the H‐ICG‐treated and folate‐blocked groups, respectively (Figure 2c,d). Overall these results demonstrate that FPH‐ICG enhances cellular uptake through specific interaction with high FRα‐expressing SK‐OV‐3 ovarian cancer cells, indicating excellent active targeting capability.

NIR‐II FLT imaging was then used to further investigate the intracellular stability of FPH‐ICG. The results showed intense signals within the cytoplasm of SK‐OV‐3 cells after incubation with FPH‐ICG for 3 h (Figure 2e,f). Quantitative analysis revealed an intracellular NIR‐II FLT of FPH‐ICG of 745 ± 2 ps, which is consistent with the inherent NIR‐II FLT of FPH‐ICG (750 ± 7 ps). Furthermore, extending the incubation time of FPH‐ICG with SK‐OV‐3 cells to 6 h maintained the intracellular FLT of FPH‐ICG (Figure 2g), it demonstrated that ICG remains tightly bound to albumin for at least 6 h following cellular internalization, and that FPH‐ICG does not undergo significant degradation. The above results indicate that FPH‐ICG can be efficiently internalized by SK‐OV‐3 cells through the interaction of folic acid with the highly expressed FRα on the cell surface and can be stably localized in the cytoplasm of SK‐OV‐3 cells for an extended period.

### 2.3 Active‐Targeting NIR‐II FL Imaging In Vivo

**Figure 3:**
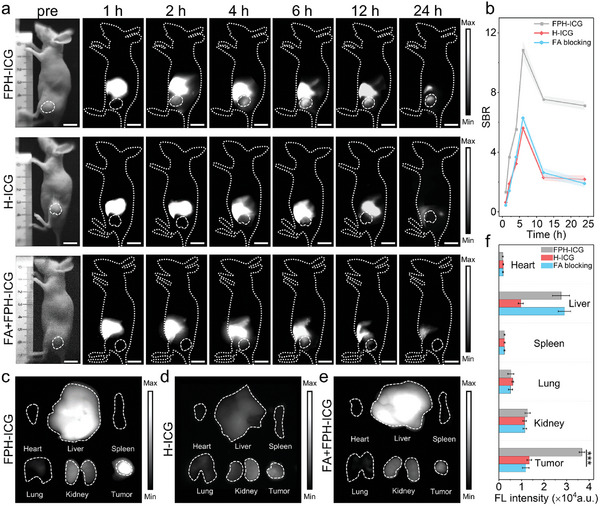
In vivo NIR‐II FL imaging of FPH‐ICG in SK‐OV‐3 subcutaneous tumor‐bearing mice. a) In vivo NIR‐II FL imaging of SK‐OV‐3 tumor‐bearing mice at various time points in the FPH‐ICG‐treated group, H‐ICG‐treated group, and FA‐blocking group. All mice were intravenously injected with 1.5 mg kg⁻¹ of ICG. Excitation wavelength: 808 nm, 1000 nm long‐pass filter, exposure time: 50 ms, power density: 50 mW cm⁻^2^, scale bar = 1 cm. Dashed circles indicate tumor regions. b) Temporal changes in the signal‐to‐background ratio (SBR) are shown in panel a) over time (*n* = 3). c) Ex vivo FL images of tumors and major organs in the FPH‐ICG‐treated group, d) in the H‐ICG‐treated group, and e) in the FA‐blocking group at 24 h post‐injection. f) Quantitative FL analysis of various tumor tissues and normal organs as shown in panels c), d), and e). Data are presented as mean ± SD (*n* = 3). ***p < 0.001.

Inspired by the promising in vitro active targeting, we further investigated the imaging performance of FPH‐ICG in vivo. SK‐OV‐3 tumor‐bearing mouse models were used to evaluate the performance of active targeting NIR‐II FL imaging. The mice were divided into three groups: 1) the FPH‐ICG‐treated group, 2) the H‐ICG‐treated group, and 3) the FA‐blocking group (**Figure** **3**a). The results showed that the tumor area in the FPH‐ICG‐treated group showed FL shortly after intravenous injection, as early as 2 h post‐injection, significantly earlier than those in the H‐ICG‐treated group. Furthermore, the FL signal in the tumor regions of the FPH‐ICG‐treated group peaked at 6 h post‐injection, with a signal‐to‐background ratio (SBR) of 10.8 far exceeding the ratio of 6.1 observed in the H‐ICG‐treated group. In the FA‐blocked group, the FL signal in the tumor regions decreased significantly compared to the FPH‐ICG‐treated group at 6 h post‐injection (Figure 3b). This suggests that FPH‐ICG enhances its in vivo targeting ability primarily by targeting the overexpressed surface folate receptors on SK‐OV‐3 cells.

One day after injection the tumor‐bearing mice were euthanized and the major organs including heart, liver, spleen, lung, kidney, and tumors were collected for ex vivo NIR‐II FL imaging (Figure 3c–e). The results showed a predominant distribution of FPH‐ICG and H‐ICG in liver and tumor tissues, suggesting that the metabolic pathways of ICG loaded within the albumin hydrophobic cavities remained unchanged.^[^ ^21^ ^]^ Furthermore, the FL signal within the excised tumor tissues from the FPH‐ICG‐treated group was 2.8 times higher than that observed in the H‐ICG‐treated group (Figure 3f), further highlighting the effective tumor‐targeting ability of FPH‐ICG.

### 2.4 NIR‐II FLT Imaging of Tumor Margins In Vivo

**Figure 4:**
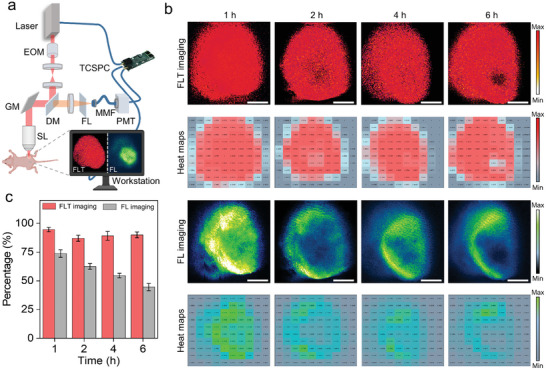
In vivo NIR‐II FLT and FL imaging of the SK‐OV‐3 subcutaneous tumor mouse model. a) Schematic representation of the custom‐built microscopy system for NIR‐II FLT and FL imaging of the mouse model. b) FLT (red) and FL (green) images of the tumor at various time points after the intravenous FPH‐ICG injection, along with heat maps of normalized FLT and FL calculated within a 10 × 10 grid (excitation wavelength: 808 nm; 1000 nm long‐pass filter; exposure time: 15 ms; power density: 40 mW cm⁻^2^). c) Ratio of positive regions in FLT and FL imaging of subcutaneous mouse tumors at 1, 2, 4, and 6 h relative to histopathologically validated positive regions. Scale bar = 2 mm.

Given the unique advantages of in vitro NIR‐II FLT imaging and the effective in vivo active targeting of FPH‐ICG, we performed quantitative investigations of its application for tumor margin visualization using a custom‐built NIR‐II mesoscopic FLT imaging system with a 7.5 × 7.5 mm^2^ field of view (max), 250 ps instrument response function (IRF) and high 64 ps time resolution (**Figure** **4**a).^[^ ^22^ ^]^ As shown in Figure 4b, clear SK‐OV‐3 tumor margins were observed 1 h after the FPH‐ICG intravenous injection. This observation was consistent with histopathological analysis by hematoxylin and eosin (H&E) staining (Figure S10, Supporting Information), confirming the high accuracy of NIR‐II FLT imaging in delineating tumor margins. Furthermore, this result remained consistent for up to 6 h, allowing precise surgical resection of tumor tissue. In contrast, NIR‐II FL imaging is dependent on the local concentration of FPH‐ICG, leading to a heterogeneous fluorescence intensity distribution. In addition, NIR‐II FL imaging of tumor margins showed temporal variability that was inconsistent with margins identified by pathological examination. Using H&E‐stained pathology images as the gold standard, NIR‐II FLT imaging visualized the SK‐OV‐3 tumor margins with over 90% accuracy, whereas NIR‐II FL imaging achieved only 58% accuracy within 6 h of the intravenous injection (Figure 4c). These findings highlight how variations in FPH‐ICG uptake by ovarian cancer cells can affect the accuracy of NIR‐II FLT imaging for delineating tumor margins.

### 2.5 NIR‐II FLT Imaging of Clinical Tumor Margins

**Figure 5:**
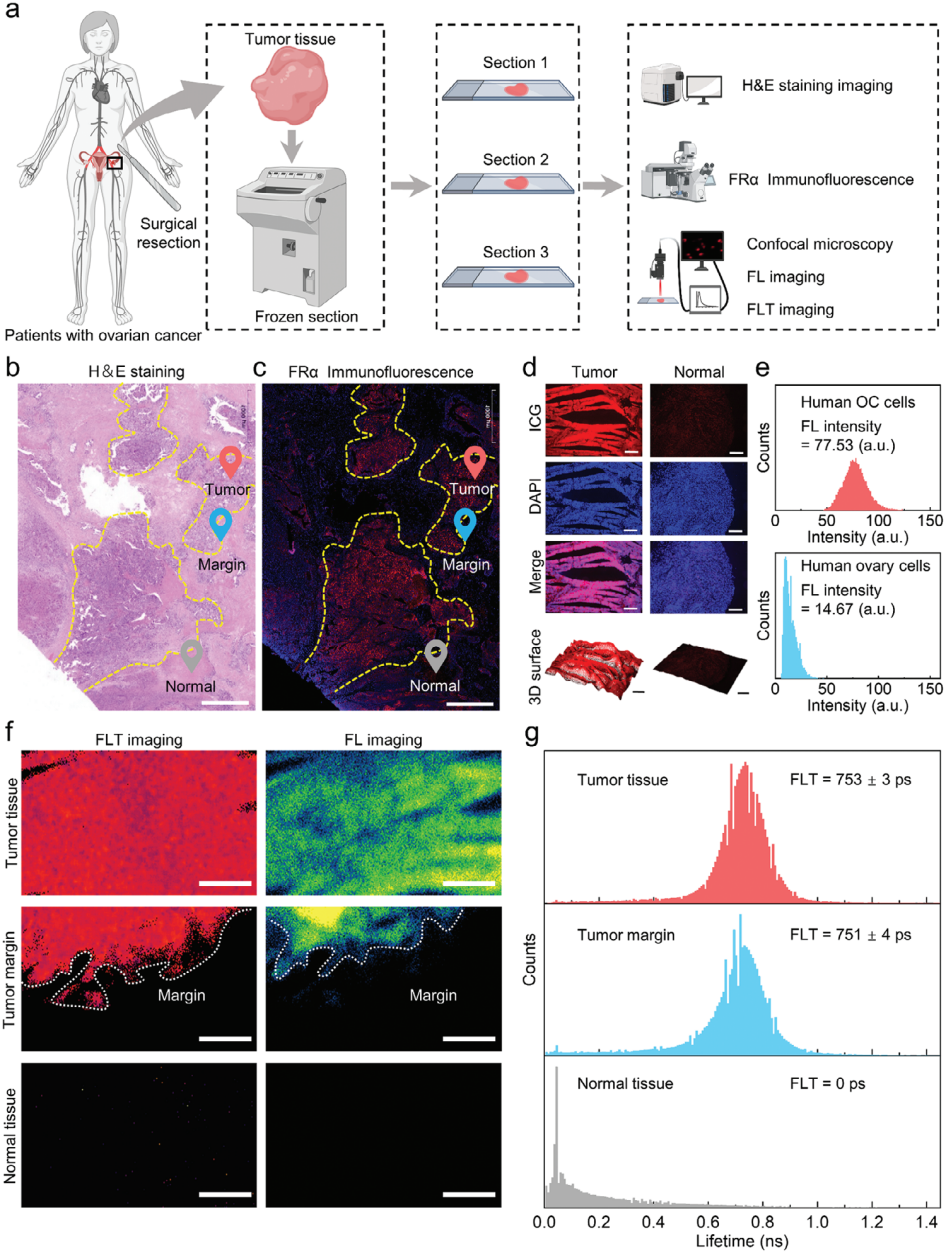
NIR‐II FLT imaging of clinical tumor margins. a) Schematic representation of the frozen sectioning and imaging process for surgically resected tumor tissue in patients with ovarian cancer. b) H&E staining results of tumor tissue. Scale bar = 1 mm. c) Specific immunofluorescence staining results for FRα in tumor tissue. Scale bar = 1 mm. d) Confocal FL imaging of tumor and normal cells after 30 min of incubation with FPH‐ICG, including 3D surface mapping of the ICG channel. ICG concentration: 40 µg mL⁻¹; red: ICG, blue: DAPI; scale bar = 300 µm. e) Histogram showing the FL intensity distribution between tumor cells and normal cells as depicted in d). f) NIR‐II FLT and FL imaging of tumor tissue, tumor margins, and normal tissue after 30 min of incubation with FPH‐ICG in the dark at room temperature. ICG concentration: 40 µg mL⁻¹; excitation wavelength: 808 nm, 1000 nm long‐pass filter, power density: 0.53 mW cm⁻^2^; scale bar = 150 µm. g) Histogram showing the FLT distribution in tumor tissue, tumor margins, and normal tissue as depicted in f).

We further investigated the ability of FPH‐ICG for NIR‐II FLT imaging of clinical tumor margins. Fresh tumor tissue samples were collected from patients who underwent resection at Renji Hospital Affiliated to Shanghai Jiao Tong University School of Medicine (this procedure follows ethical standards and has been approved by the Medical Ethics Committee of Renji Hospital Affiliated to Shanghai Jiao Tong University School of Medicine (2018‐114‐CR‐05)) and immediately processed into 5 µm thick frozen sections for H&E staining, FRα immunofluorescence imaging, confocal FL imaging, and NIR‐II FLT imaging, respectively (**Figure** **5**a). Pathological analysis confirmed that the patient had FRα‐positive ovarian cancer (Figure 5b,c). FL confocal microscopy imaging confirmed that FPH‐ICG could specifically detect clinical tumor cells and normal cells with an SBR of 5.3 (Figure 5d,e). We then selected the pathologically confirmed tumor margins for NIR‐II FLT imaging (Figure 5f). The tumor regions exhibited bright NIR‐II FLT signals of 753 ± 3 ps, whereas no NIR‐II FLT signals (0 ps) or FL signals were detected in the normal tissue regions. Tumor margins were delineated by both NIR‐II FLT and FL imaging. Compared to NIR‐II FL imaging, NIR‐II FLT imaging was able to detect the infiltrative tumor margins (751 ± 4 ps), which was consistent with the pathological imaging results (Figure 5g). These results confirm that FPH‐ICG can specifically target FRα‐positive ovarian cancer cells and achieve accurate NIR‐II FLT imaging of tumor margins, demonstrating its potential for intraoperative navigation in ovarian cancer surgery.

### 2.6 NIR‐II FLT Imaging‐Guided Surgery In Vivo

**Figure 6:**
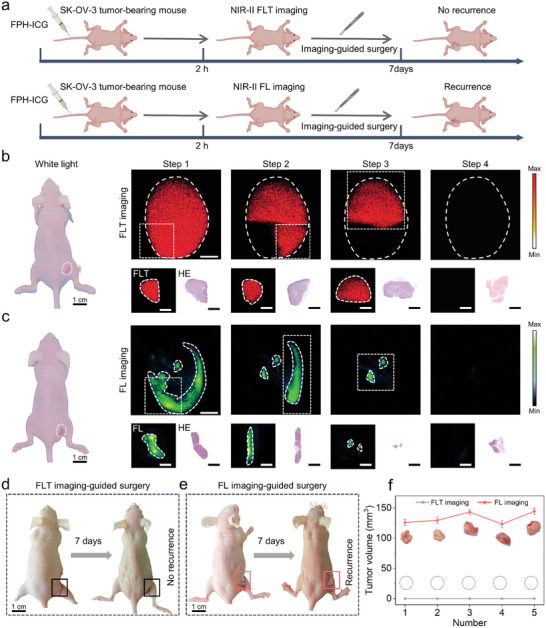
In vivo NIR‐II FLT image‐guided surgery. a) Schematic representation of the FLT and FL image‐guided surgeries. b) White light imaging of mice and FLT image guided step‐by‐step tumor resection, followed by FLT imaging and H&E staining of the resected tissue. Scale bar = 1.5 mm. c) White light imaging of mice and FL image guided step‐by‐step tumor resection, followed by FL imaging and H&E staining of the resected tissue. Scale bar = 1.5 mm. d) Tumor recurrence observed following surgery guided by FLT imaging. e) Tumor recurrence was observed following surgery guided by FL imaging. f) FLT and FL images illustrating recurrent tumors after surgical guidance (*n* = 5).

Inspired by the excellent active targeting capability of FPH‐ICG in vivo and its precise NIR‐II FLT imaging capabilities, we evaluated the potential of using NIR‐II FLT imaging of FPH‐ICG to guide the surgical resection of SK‐OV‐3 in tumor‐bearing mice (**Figure** **6**a). When the tumor volume reached ≈100 mm^3^, we performed NIR‐II FLT image‐guided animal surgical resection experiments 2 h after the intravenous injection to simulate clinical surgical scenarios. The operation followed the ethical code and was approved by the Animal Care and Use Committee of the Shenzhen Institutes of Advanced Technology (SIAT), Chinese Academy of Sciences (SIAT‐IACUC‐20210514‐YGS‐YXZX‐SZH‐A0329‐01‐03). NIR‐II FLT imaging of FPH‐ICG accurately delineated the tumor boundaries, facilitating stepwise tumor excision. Histopathological analysis using H&E staining confirmed the removal of cancerous tissue in steps 1, 2, and 3. No NIR‐II FLT signal was detected at the incision site after step 3, indicating complete tumor removal. Tissue taken from the surgical margins for H&E staining showed normal tissue, confirming the thorough excision guided by NIR‐II FLT imaging (Figure 6b). In contrast, NIR‐II FL imaging was unable to fully delineate the tumor contours. These findings indicate that while NIR‐II FL imaging could be used to partially guide tumor removal (steps 1–3), the H&E staining showed that this technique did not remove the full tumor (Figure 6c). Tumor volume measurements showed no recurrence in the NIR‐II FLT image‐guided surgery group. Conversely, significant tumor recurrence was observed in all 5 mice in the NIR‐II FL image‐guided surgery group (Figure 6d,e), with each mouse developing tumor volumes >100 mm^3^ (Figure 6f). These findings underscore the superior accuracy of NIR‐II FLT with FPH‐ICG image‐guided surgical resection compared to NIR‐II FL imaging. It should be noted that, whether guided by FLT imaging or FL imaging, there is a slight decrease in mouse body weight after surgery (Figure S11, Supporting Information). The integration of NIR‐II FLT imaging technology with active targeting nanoprobes promises to improve the accuracy of surgical tumor delineation. However, further clinical studies are required to confirm its utility clinically.

## 3 Conclusion

In conclusion, the engineered FRα‐targeted FPH‐ICG nanoprobes developed in this study significantly improved the precision of tumor margin delineation of NIR‐II FLT imaging. The remarkable affinity of FPH‐ICG for FRα‐expressing ovarian cancer cells, combined with its extended NIR‐II FLT and enhanced NIR‐II FL intensity, facilitated precise visualization of tumor margins in both mouse models and clinical samples. Importantly, NIR‐II FLT imaging provided a more reliable and accurate depiction of tumor margins compared to conventional NIR‐II FL imaging, as confirmed by histopathological analysis. These results highlight the promise of FPH‐ICG nanoprobes in guiding precise surgical interventions via NIR‐II FLT imaging, offering a promising strategy to improve surgical outcomes in ovarian cancer therapy.

## 4 Experimental Section

### Materials

Fetal bovine serum (FBS) and phosphate‐buffered saline (PBS) (pH 7.4) were purchased from Gibco (Switzerland). McCoy's 5A medium and penicillin‐streptomycin solution were purchased from Pricella Biotechnology (Wuhan, China). ICG, N‐(3‐dimethylaminopropyl)‐N'‐ethylcarbodiimide hydrochloride (EDC), N‐hydroxysuccinimide (NHS) and human serum albumin (HSA) were purchased from Sigma‐Aldrich (USA). The H&E staining kit was purchased from Solarbio (Beijing, China). In addition, FA‐PEG_2000_ was purchased from Pengsheng Biological (Shanghai, China).

### Molecular Docking

HSA has three drug‐binding sites (site‐I, site‐II, site‐III). To accurately identify the binding sites of ICG on HSA, HSA crystal structures (Apo) was used that did not bind to any ligands, site‐I bound to warfarin (1h9z), site II bound to dansyl‐L‐norvaline (2xw1), and site III bound to camptothecin (4l8u) and hemoglobin (1n5u) crystal structures. Using Autodock Vina, ICG molecules were docked to each of the three HSA drug sites. The free binding energies of each drug site were compared to identify the most probable binding site and conformation.

The initial small molecule structures (site‐1, site‐3) selected in this article were derived from the HSA‐ICG complex calculated by docking and subjected to hydrogenation treatment using GaussView6.0 software. The small‐molecule structure was determined using B3LYP‐D3 density functional theory (DFT). The 6–31G(d) basis set was employed for both single‐point energy and geometry optimization calculations. Additionally, zero‐point energy correction was applied. The excited state calculation was performed using TD‐DFT and the same basis set calculation method. All calculations were performed using the Gaussian 16 program.

### Preparation of H‐ICG and FPH‐ICG

The ICG (0.25 mg mL⁻¹) aqueous solution was mixed with HSA (10 mg mL⁻¹) in pure water at 4 °C for 12 h. The mixed solution was then ultrafiltered at 8000 rpm for 30 min using an ultrafiltration tube (MWCO, 30 kDa) to remove the free ICG solution. During this process, ICG and HSA docked to form the H‐ICG nanoparticles (NPs). Subsequently, FPH‐ICG was prepared by adding an aqueous solution of carboxylated FA‐PEG_2000_ (1.5 mg mL⁻¹) activated with 1‐Ethyl‐3‐(3‐dimethylaminopropyl) carbodiimide (EDC) (0.4 mg mL⁻¹) and N‐Hydroxysuccinimide (NHS) (0.42 mg mL⁻¹) in pure water at 4 °C for 4 h. The active FA‐PEG_2000_ was further reacted with H‐ICG for 24 h. The mixture was then ultrafiltered at 8000 rpm for 30 min using an ultrafiltration tube (MWCO, 30 kDa) to remove the free FA‐PEG_2000_ and obtain the FPH‐ICG solution.

### Characterization

UV–vis–NIR spectrophotometer was used to characterize the ultraviolet‐visible–near‐infrared (UV–vis–NIR) absorbance spectra of FPH‐ICG, HSA, and FA‐PEG_2000_ (HSA: 1 mg mL^−1^, FA‐PEG_2000_: 0.15 mg mL^−1^) in the range of 240–515 nm using the (Shimadzu, Japan). In addition, UV–vis–NIR absorption spectra of H‐ICG, FPH‐ICG, and free ICG at the same ICG concentration in the range of 600–900 nm were also characterized. The NIR‐I and NIR‐II FL emission spectra were obtained using an FS980 FL spectrometer (Edinburgh Instruments, UK) were obtained using an excitation wavelength of 710 nm, FL emission of 730 nm (NIR‐I), an excitation wavelength at 808 nm, and a 1000 nm long‐pass filter (NIR‐II). The hydrodynamic sizes of HSA, H‐ICG, and FPH‐ICG were determined by dynamic light scattering (DLS) using the Nano‐ZS90 (Malvern Instruments, UK).

The morphology of the NPs was observed by atomic force microscopy (AFM) (Shimadzu spm9700, Japan) in tapping mode. For this analysis, an aqueous solution of FPH‐ICG NPs (10 mg mL^−1^) was used, and a 30 µL NP sample was dropped onto a freshly cleaved mica substrate (1 cm^2^) and air‐dried at room temperature for 12 h. During imaging, a force of 42 N m^−1^ was applied to the cantilever tip at a frequency of 1 Hz. The resulting AFM NPs images were processed using the Gwyddion software.

### Time‐Resolved NIR‐II Imaging System

The fluorescent lifetime imaging microscopy (FLIM) method used in this study was based on the classical time‐correlated single photon counting (TCSPC) system. The imaging process was carried out on a custom‐built in vivo NIR‐II mesoscopic microscope (sketched in Figure 4a). A Sapphire femtosecond laser (Ultra I, Coherent) capable of emitting pulses at a wavelength ranging from 690 to 1040 nm, 200 ps, and an 80 MHz repetition rate was used as the excitation source. For this study, a wavelength of 808 nm was selected to excite the FPH‐ICG according to the manufacturer's recommendations. The laser beam was adjusted to the optimum excitation power using an EOM (350‐80‐LA‐02, Conoptics). The laser was then passed through a 4‐f system to function as a beam expander before entering the DM (DMLP1000, Thorlabs). After being reflected by the DM, the laser beam was scanned by the GMs and propagated to the SL (LSM02‐BB, Thorlabs). To achieve a large field of view, the SL was used instead of a traditional lens to focus the laser on the sample. The FL was filtered by a long‐pass filter and collected by a multimode fiber. The FL was detected by a NIR PMT (H12397‐75, Hamamatsu). Finally, the laser synchronization signal, detector pulse, and GM synchronization signals were recorded by a TCSPC (FT1040, SIMINICS Inc). The FL decay curve was modeled by a monoexponential function f(t) = ae^(‐t/τ), where τ represented the lifetime of the dye. The lifetime was calculated by fitting the f(t) obtained by TCSPC.

### Cell Culture

SK‐OV‐3 ovarian cancer cells (catalog number: SCSP‐5214) were obtained from the Shanghai Cell Bank, Chinese Academy of Sciences. The cells were cultured under sterile conditions at 37 °C and 5% carbon dioxide in McCoy's 5A medium supplemented with 10% FBS and 1% Pen‐Strep. Trypsin was then added to digest the cells, and PBS buffer was used to wash the cells. All procedures related to cell recovery, passaging, and cryopreservation were conducted in a sterile environment.

### Cellular Uptake

The cellular uptake of FPH‐ICG NPs was investigated by seeding SK‐OV‐3 cells (1 × 10⁵) in glass‐bottom culture dishes. After 12 h, the cells were cultured with FPH‐ICG NPs at 37 °C for 3 to 6 h, washed three times with PBS at room temperature, and fixed with 4% polyformaldehyde. Cellular FLT imaging was then performed using the custom‐built NIR‐II mesoscopic FLT imaging system (excitation: 808 nm; long pass: 1000 nm; exposure time: 15 ms). In a parallel setup, cells were seeded in the same manner and, after 12 h, incubated with three different NP samples (FPH‐ICG, H‐ICG, or FPH‐ICG NPs in the presence of excess FA as a competitor and ICG concentrations of 25 µg mL^−1^) at 37 °C for 3 h. These samples were then observed using a confocal laser scanning microscope (CLSM, Nikon). In addition, active targeting analysis of cells to the three types of NPs was performed using a CytoFLEX flow cytometer (Beckman Coulter, USA). The data was quantitatively analyzed using FlowJo software (*n* = 3).

### Processing and Imaging of Clinical Samples

Surgical resection of tumor tissue was performed on patients with ovarian cancer (*n* = 2). The samples were collected at Renji Hospital affiliated with Shanghai Jiao Tong University School of Medicine, and all patients in the study gave informed consent for their participation in the study. This study was approved by the Medical Ethics Committee of Renji Hospital Affiliated to Shanghai Jiao Tong University School of Medicine (2018‐114‐CR‐05). After freezing tissues in −80 °C, 5‐µm sections were cut from the frozen block. Then the frozen sections were incubated with FPH‐ICG (30 µg mL^−1^) for 30 min in a dark at room temperature, washed with PBS three times, and stained with DAPI for 5 min. Subsequently, the sections were imaged with a confocal laser scanning microscope and the custom‐built NIR‐II mesoscopic FLT imaging system described above.

Frozen sections were also subjected to H&E staining and immunofluorescence (IF) staining. To retrieve the antigen, the slides were autoclaved in EDTA antigen retrieval buffer (pH 6.0) for ≈15 min, washed with PBS three times, then treated with 5% goat serum for 30 min to block non‐specific binding. The slides were then incubated with FBP antibody (A15672, ABclonal, 1:100) at 4 °C overnight, washed with PBS three times, and then incubated with secondary antibody (A‐11011, Invitrogen, 1:100) for 50 min at room temperature. Finally, the slides were washed with PBS three times and incubated with DAPI solution at room temperature for 5 min in the dark.

### Tumor Models

Female BALB/c nude mice weighing between 18 and 22 g and aged between 6 and 8 weeks were obtained from Zhuhai Bestest Biotechnology Co., Ltd. (China) and housed in a sterile environment at room temperature, following a 12‐h light/dark cycle. The mice were provided with unrestricted access to food and water. All animal experiments were approved by the Animal Care and Use Committee of the Shenzhen Institutes of Advanced Technology (SIAT), Chinese Academy of Sciences (SIAT‐IACUC‐20210514‐YGS‐YXZX‐SZH‐A0329‐01‐03). The mice were then anesthetized with an intraperitoneal (IP) injection of avertin (2 mL kg^−1^), and a subcutaneous tumor model was established by injecting SK‐OV‐3 cells (1 × 10^6^) into the left flank of the mice. After 7 to 10 days, the mice with tumor volumes above 100 mm^3^ were identified and used for the subsequent experimental studies.

### In Vivo NIR‐II FL Imaging

The subcutaneous SK‐OV‐3 tumor‐bearing nude mice were injected intravenously with either FPH‐ICG or H‐ICG NPs (1.5 mg kg⁻¹ ICG). FL images were then acquired using a NIR‐II FL imaging system (NIRvana 640, Teledyne Princeton Instruments, USA) equipped with an 808 nm laser and a 1000 nm long pass filter (excitation: 808 nm; long pass: 1000 nm; exposure time: 100 ms). The mice (*n* = 3). were euthanized 24 h after injection and the major organs were harvested. NIR‐II FL images of the organs were then obtained using the same imaging parameters described above.

### NIR‐II FLT Imaging In Vivo

The mice (*n* = 3) were anesthetized using 1 to 2% (vol/vol) isoflurane in oxygen. FPH‐ICG NPs (1.5 mg kg⁻¹ ICG) were injected intravenously into the subcutaneous SK‐OV‐3 tumor mouse model. In vivo, NIR‐II FLT imaging was performed using a custom‐built optical experimental platform (Shenzhen Institutes of Advanced Technology, Chinese Academy of Sciences) (excitation: 808 nm; long pass: 1000 nm; exposure time: 15 ms).

### Image‐Guided Surgery In Vivo

FPH‐ICG NPs (1.5 mg kg^−1^ ICG) were intravenously injected into the SK‐OV‐3 tumor‐bearing BALB/c nude mice. After 6 h of dosing, the mice were anesthetized by intraperitoneal (IP) injection of Avertin (2 mL kg^−1^) to reduce excessive pain during surgery. FLT and FL imaging of SK‐OV‐3 tumors was then performed using a custom‐built optical experimental platform (excitation: 808 nm; long pass: 1000 nm; exposure time: 15 ms). The gross visible tumor volume was resected under image guidance. The excised tissues were then subjected to FLT and FL imaging under the same parameters and histopathological staining. In case of positive surgical margins, additional resections were performed to remove the full tumor.

### Statistical Analysis

The continuous data are expressed as mean ± SD. The sample size for each statistical analysis was greater than or equal to 3 (n ≥ 3). The Student's t‐test (two‐tailed) was used to compare the continuous variables between the different treatment arms. A p‐value below 0.05 was deemed statistically significant. Significant difference was shown as *p < 0.05, **p < 0.01, and ***p < 0.001. Statistical analysis was carried out using GraphPad Prism 7 (GraphPad Software, Inc.) for Windows. The ImageJ software (Win 64) was used for grayscale analysis, and drew the graph using Origin Lab software (2021).

## Conflict of Interest

The authors declare no conflict of interest.

## Author Contributions

Z.C., L.H., and D.G. contributed equally to this work. Z.C., D.G., D. H., J. C., H.Z., and Z.S. conceived and designed the research, interpreted the data, and wrote the manuscript. Z.C. prepared and characterized the probes and NIR‐II imaging studies. L.H., J.L., and W.Z. calculated and processed the fluorescence lifetime. Z.B. processed and analyzed clinical tissue samples. Z.C. and Z.S. revised the manuscript. All the authors discussed and commented on the manuscript.

## Supporting information



Supporting Information

## Data Availability

The data that support the findings of this study are available from the corresponding author upon reasonable request.
