# Supplementary material for: High Spatiotemporal Near‐Infrared II Fluorescence Lifetime Imaging for Quantitative Detection of Clinical Tumor Margins
Source: Adv Sci (Weinh). 2024 Dec 9;12(5):2411272. doi: 10.1002/advs.202411272 (PMC11791973; doi:10.1002/advs.202411272)
Supplement: Supplementary file 1 — Supporting Information [file ADVS-12-2411272-s001.docx]

Supporting Information

High Spatiotemporal Near-Infrared II Fluorescence Lifetime Imaging for Quantitative Detection of Clinical Tumor Margins

*Zhen Chen, Linjian Huang, Duyang Gao, Zhouzhou Bao*, Dehong Hu, Wei Zheng, Jing Chen*, Jiuling Liao*, Hairong Zheng, Zonghai Sheng**


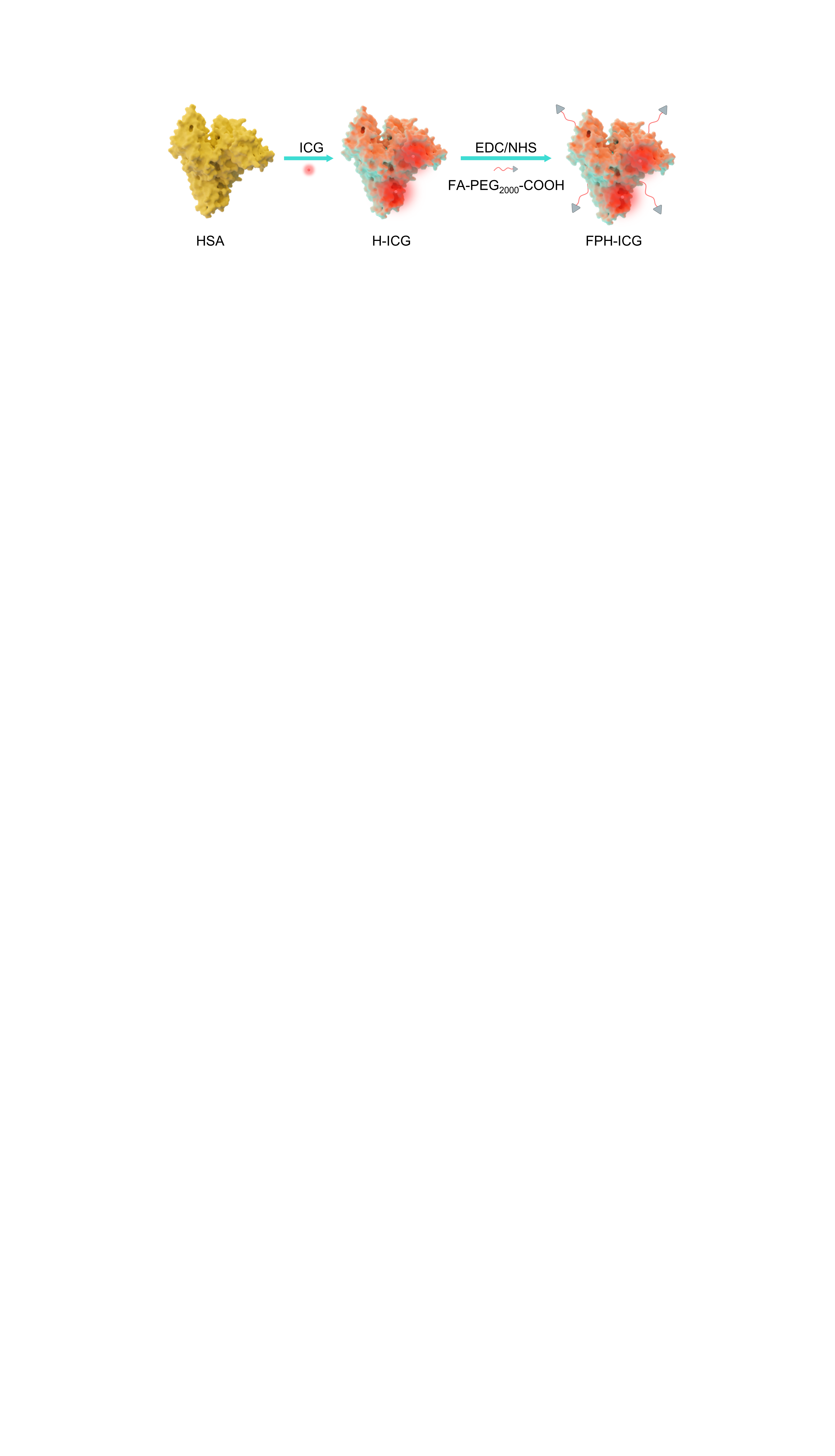


**Figure S1.** Schematic illustration of prepared process of FPH-ICG.


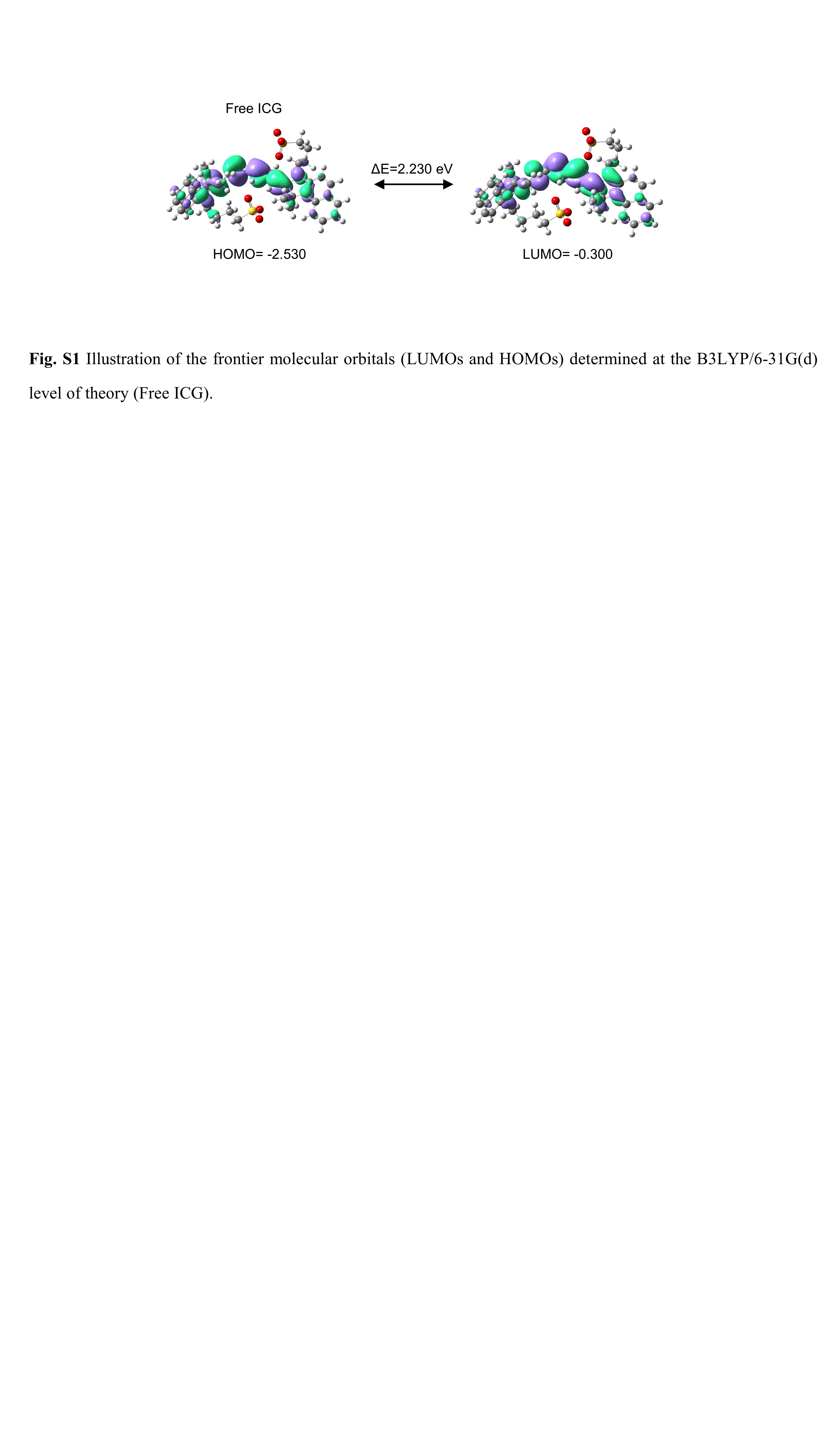


**Figure S2.** Illustration of the frontier molecular orbitals (LUMOs and HOMOs) determined at the B3LYP/6-31G(d) level of theory (Free ICG).


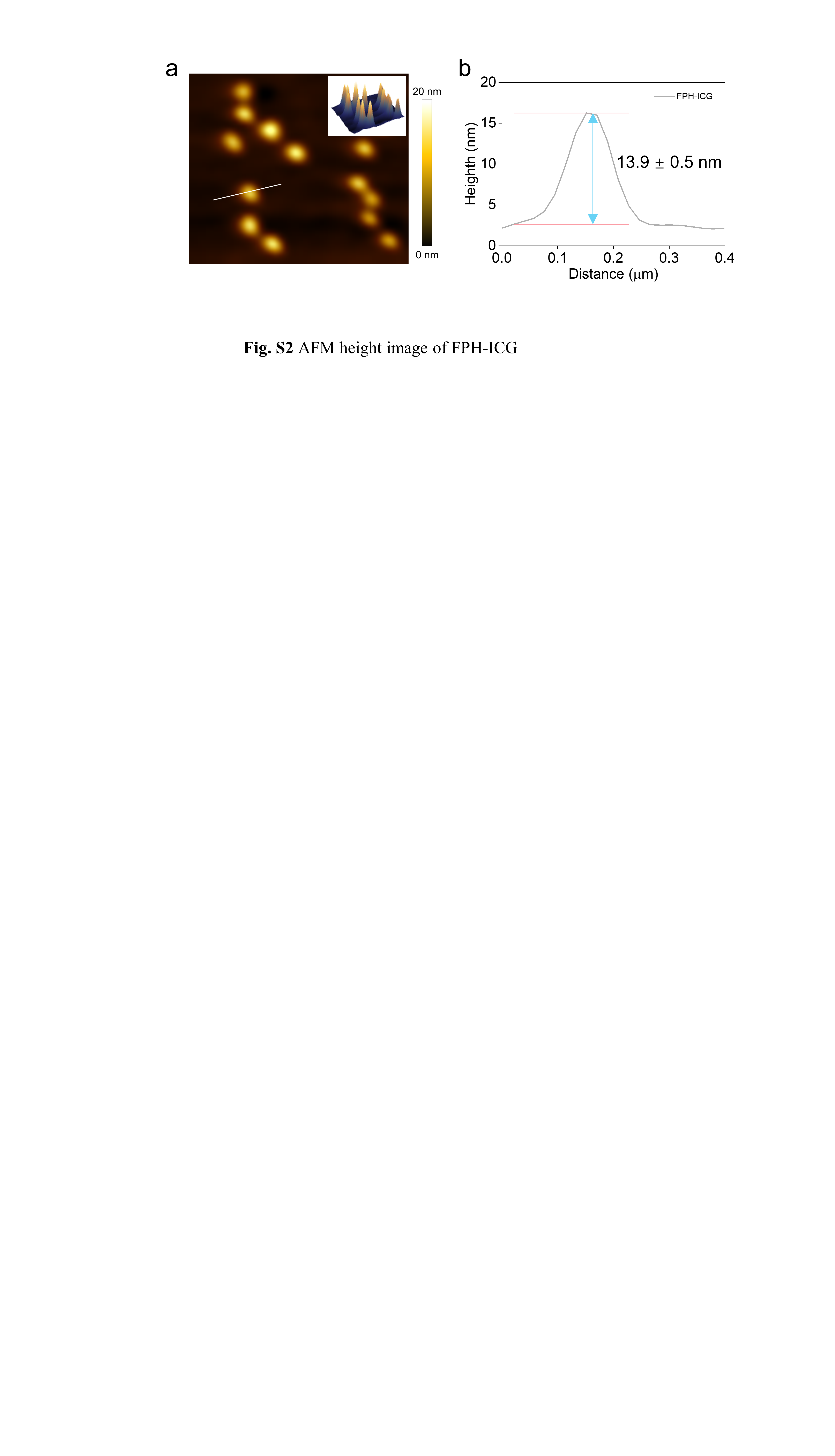


**Figure S3.** (a) AFM image and (b) height result of FPH-ICG.


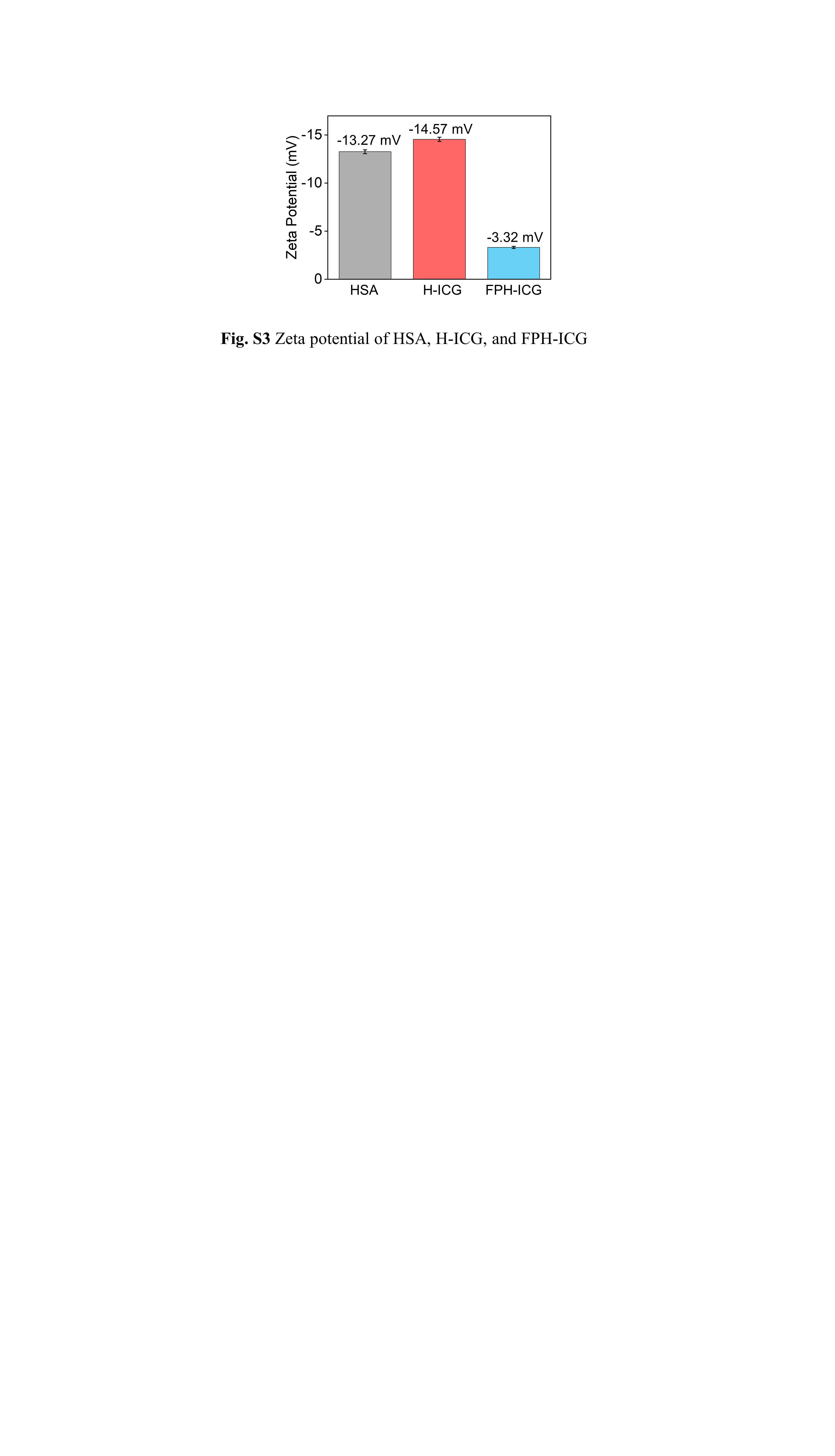


**Figure S4.** Zeta potential of HSA, H-ICG, and FPH-ICG.


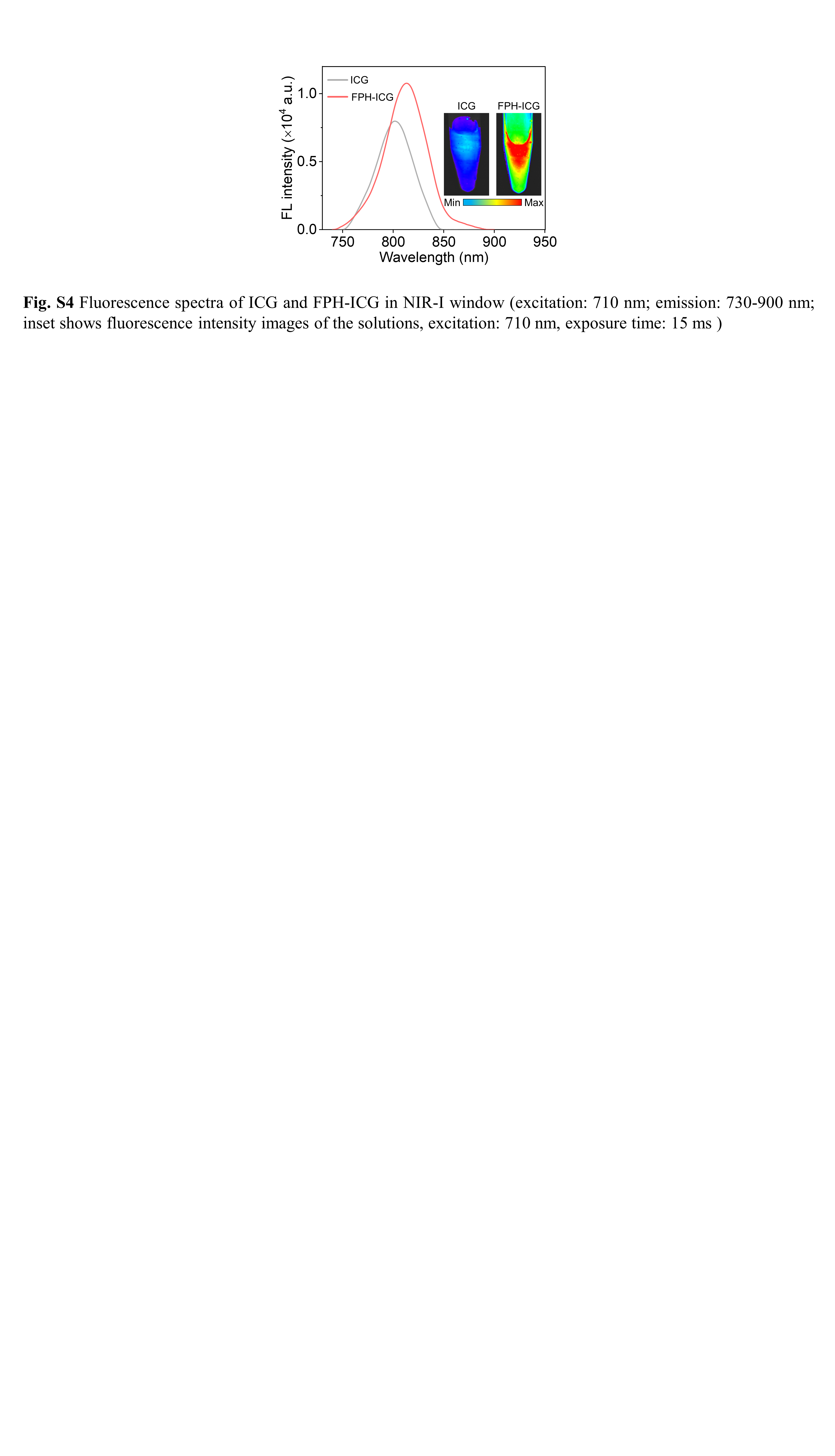


**Figure S5.** Fluorescence spectra of ICG and FPH-ICG in NIR-I window (excitation: 710 nm; emission: 730-900 nm; inset shows fluorescence intensity images of the solutions, excitation: 710 nm, exposure time: 5 ms).


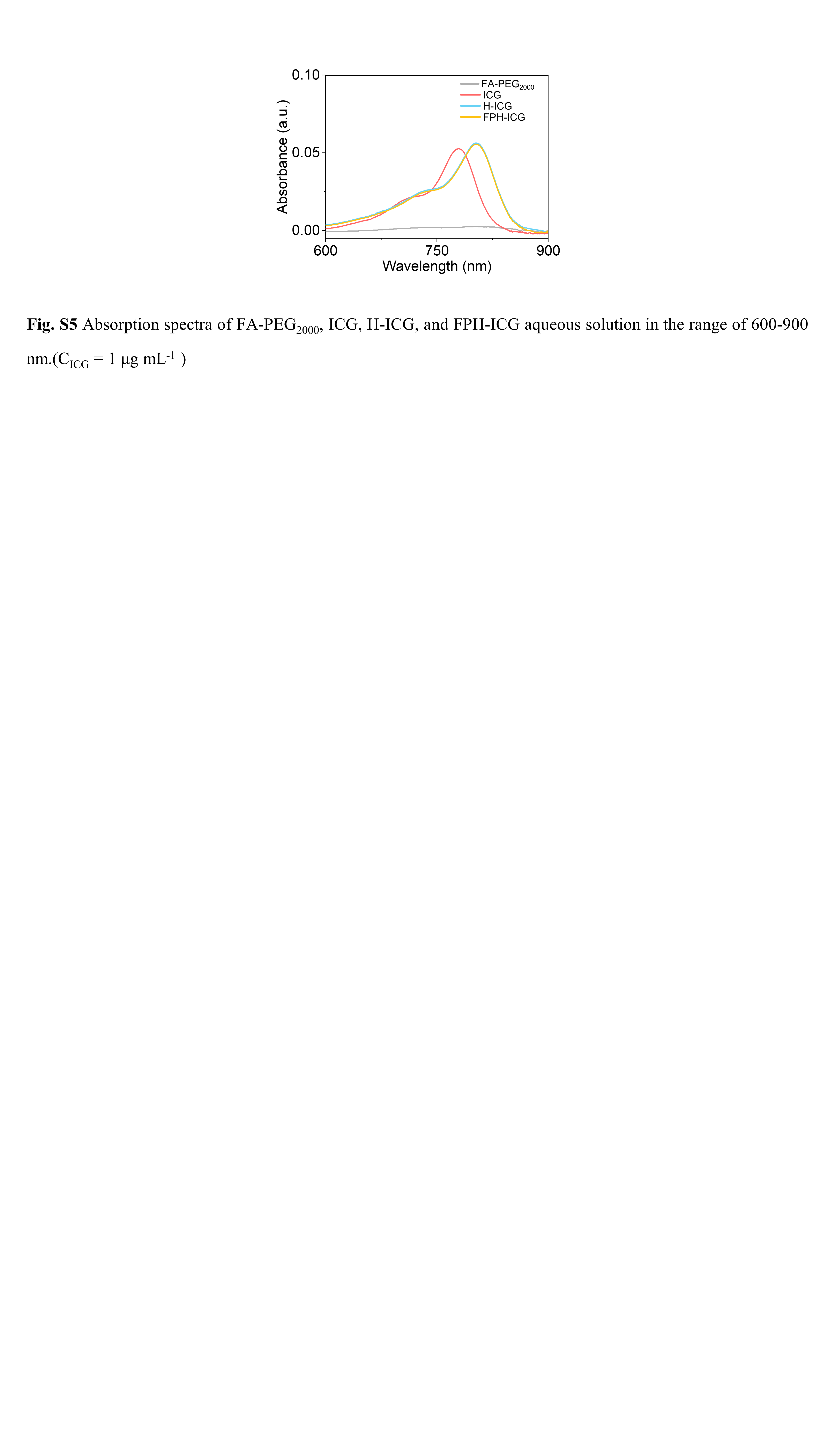


**Figure S6.** Absorption spectra of FA-PEG_2000_, ICG, H-ICG, and FPH-ICG aqueous solution in the range of 600-900 nm (C_ICG_ = 1 μg mL^-1^).


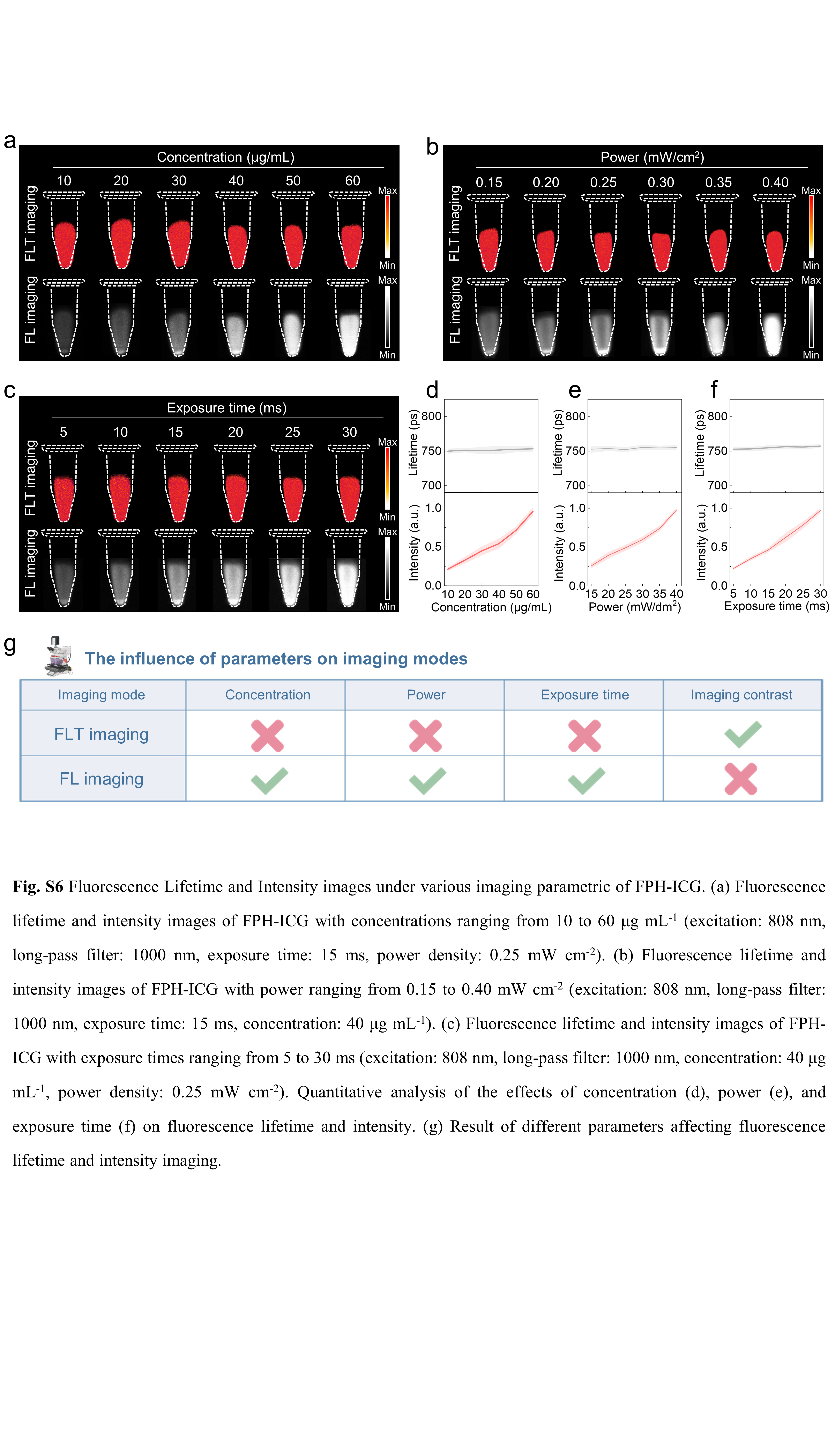


**Figure S7.** Fluorescence Lifetime and Intensity images under various imaging parametric of FPH-ICG. (a) Fluorescence lifetime and intensity images of FPH-ICG with concentrations ranging from 10 to 60 μg mL^-1^ (excitation: 808 nm, long-pass filter: 1000 nm, exposure time: 15 ms, power density: 0.25 mW cm^-2^). (b) Fluorescence lifetime and intensity images of FPH-ICG with power ranging from 0.15 to 0.40 mW cm^-2^ (excitation: 808 nm, long-pass filter: 1000 nm, exposure time: 15 ms, concentration: 40 μg mL^-1^). (c) Fluorescence lifetime and intensity images of FPH-ICG with exposure times ranging from 5 to 30 ms (excitation: 808 nm, long-pass filter: 1000 nm, concentration: 40 μg mL^-1^, power density: 0.25 mW cm^-2^). Quantitative analysis of the effects of concentration (d), power (e), and exposure time (f) on fluorescence lifetime and intensity. (g) Result of different parameters affecting fluorescence lifetime and intensity imaging.


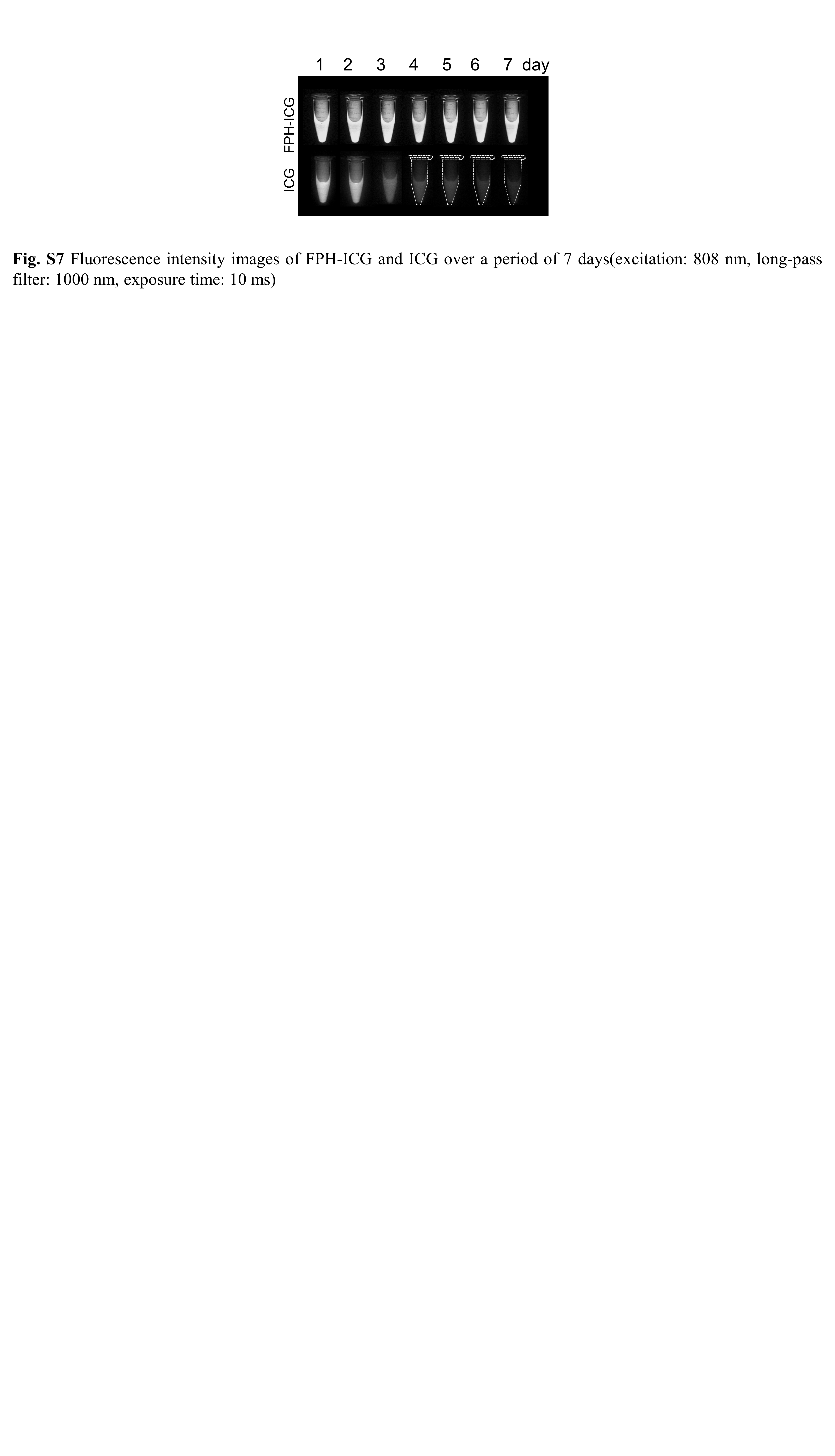


**Figure S8.** Fluorescence intensity images of FPH-ICG and ICG over a period of 7 days (excitation: 808 nm, long-pass filter: 1000 nm, exposure time: 10 ms).


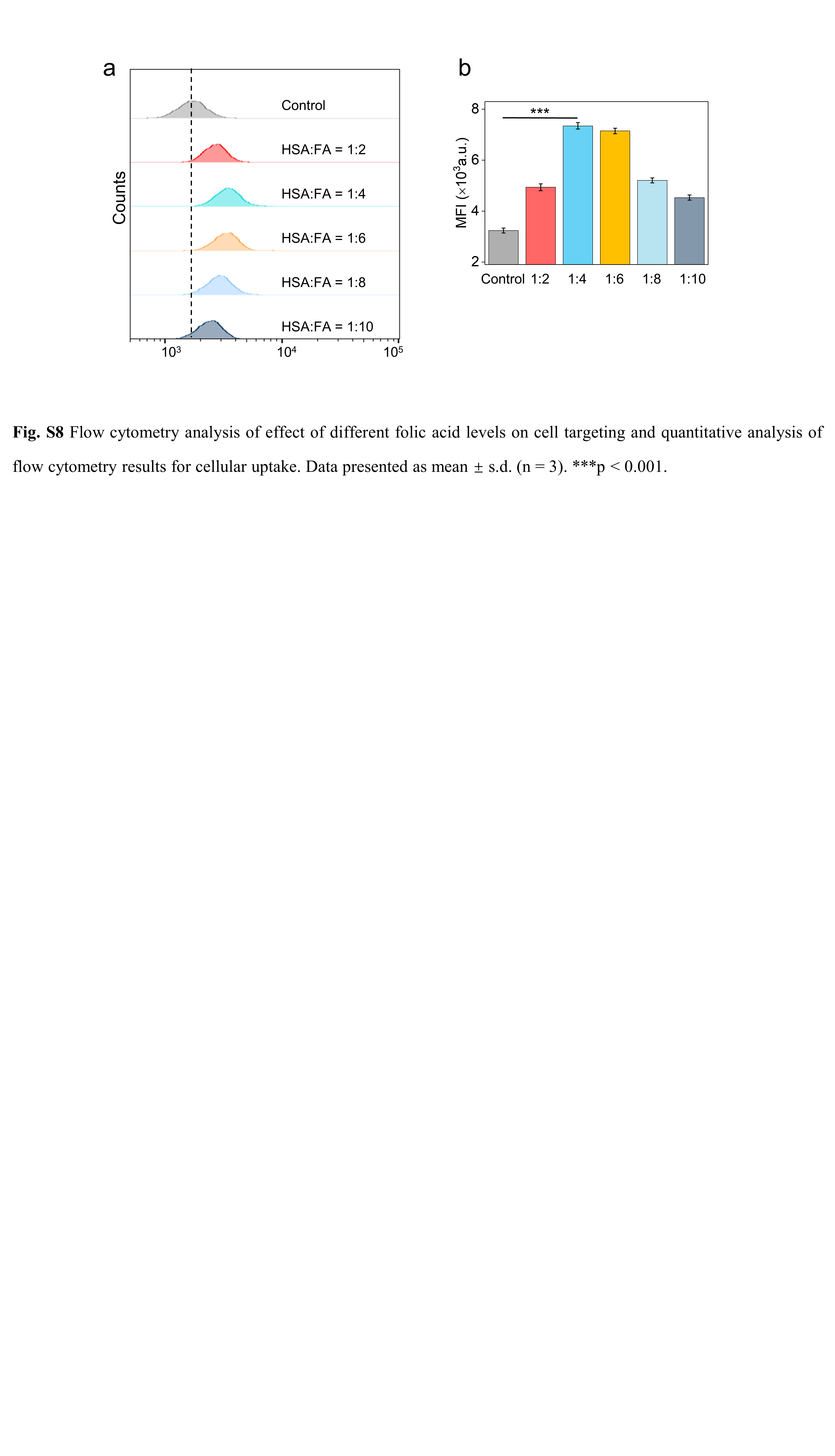


**Figure S9.** (a)Flow cytometry analysis of effect of different folic acid levels on cell targeting and (b) quantitative analysis of flow cytometry results for cellular uptake. Data presented as mean ± s.d. (n = 3). ***p < 0.001.


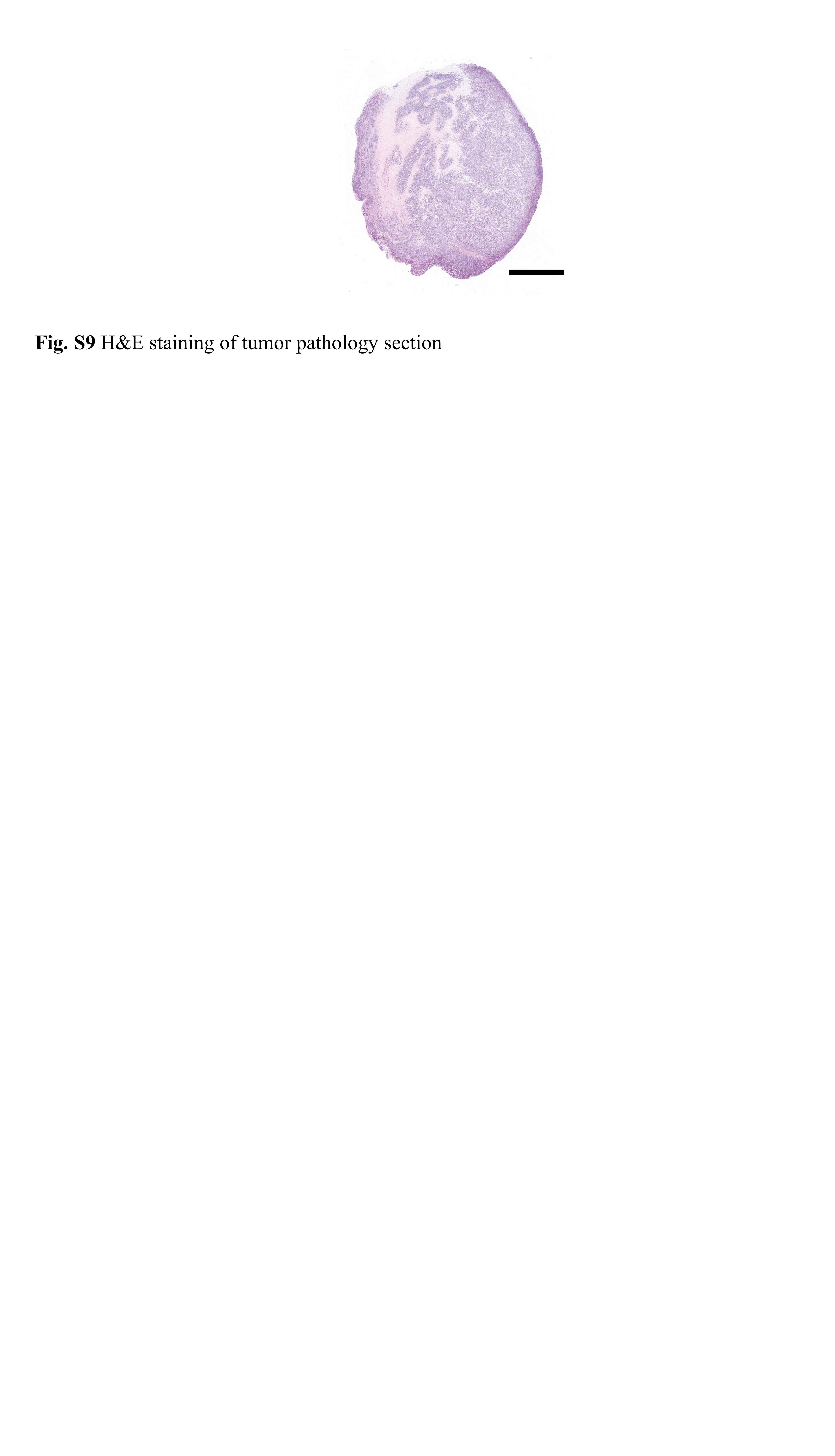


**Figure S**10**.** H&E staining of tumor pathology section. Scale bar = 2 mm.


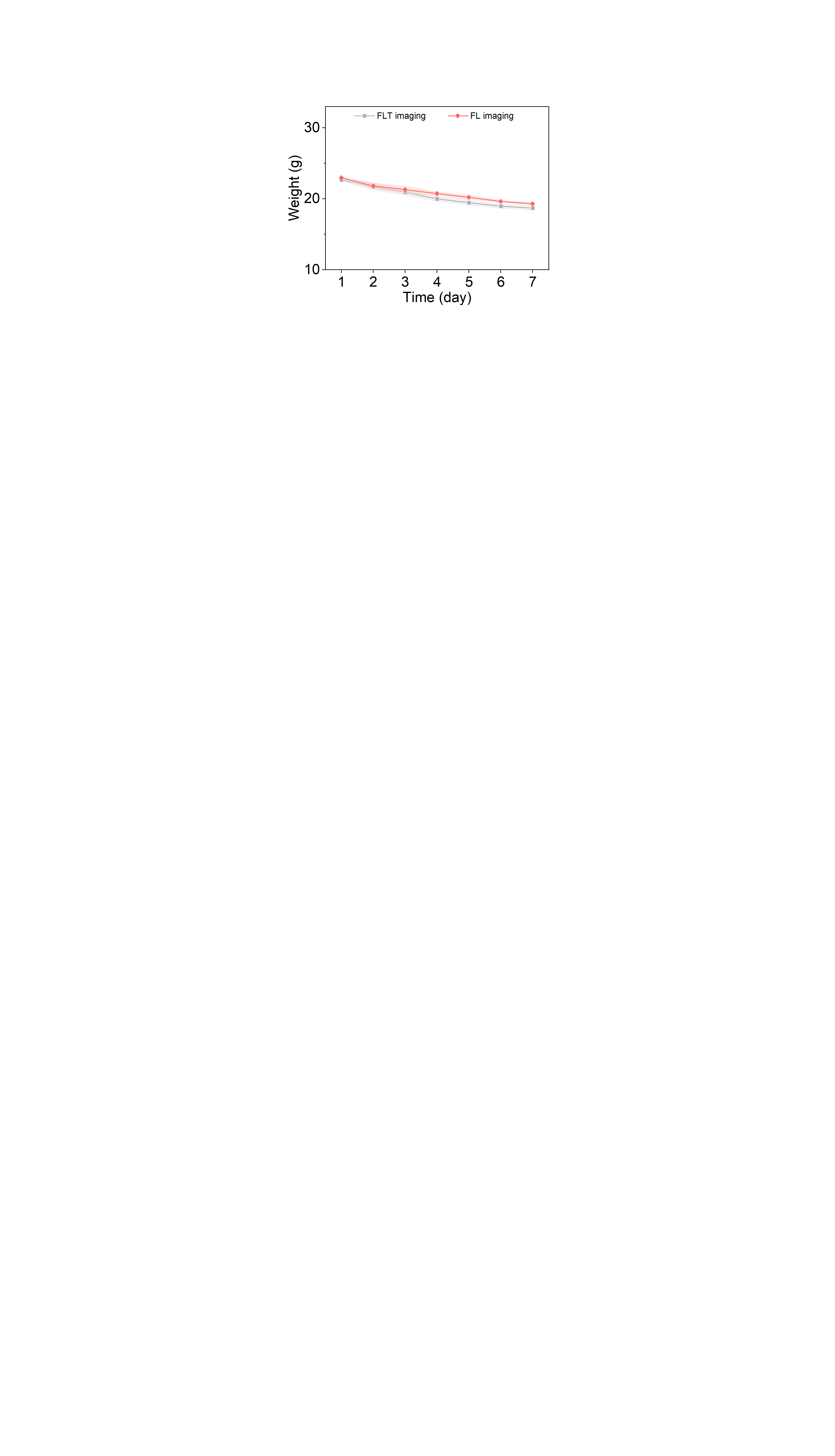


**Figure S11.** Variations in body weight of mice over a 7-day period following tumor resection, guided by fluorescence lifetime (FLT) imaging and fluorescence (FL) imaging (n = 5).
